# Authors reply in response to a letter on “impact of aminoglycosides on survival rate and renal outcomes in patients with urosepsis: a multicenter retrospective study”

**DOI:** 10.1186/s13613-025-01505-4

**Published:** 2025-09-25

**Authors:** David Rozenblat, François Dépret, Matthieu Lafaurie

**Affiliations:** 1 Service de Maladies infectieuses et Tropicales, Hôpitaux Universitaires Saint-Louis Lariboisière, Assistance Publique - Hôpitaux de Paris, Paris, F-75010 France; 2 Service d’Anesthésie-Réanimation et traitement chirurgical des grands brûlés, Hôpitaux Universitaires Saint-Louis Lariboisière, Assistance Publique - Hôpitaux de Paris, Paris, F-75010 France; 3 Sorbonne Université, Paris, France; 4 Université Paris Cité, Paris, France

Dear Editor,

We appreciate the valuable and insightful comments from Li et al. regarding our article “Impact of aminoglycosides on survival rate and renal outcomes in patients with urosepsis: a multicenter retrospective study” [1]. We also thank you for the opportunity to respond to their concerns, which have helped improve the clarity and quality of our work. The following is our author response.

First, we fully acknowledge the limitations of SCr as a biomarker. It is indeed well known that SCr may underestimate early or mild acute kidney injury (AKI), particularly in critically ill patients. However, SCr remains the internationally accepted reference standard for defining AKI according to Kidney Disease Improving Global Outcomes (KDIGO) criteria, and it is the only biomarker that is routinely used and retrospectively available in hospital databases [2]. The retrospective design of our study led us retain SCr as the primary marker. Importantly, we included a 30-day follow-up and assessed robust clinical endpoints, including renal recovery, new renal replacement therapy and the Major Adverse Kidney Events at day 30 (MAKE 30) criteria. None of these criteria showed significant differences between groups. Moreover, only 3.6% of patients without AKI at baseline developed AKI during follow-up. While we recognize that this does not rule out subclinical or mild AKI, our findings argue against a major undetected nephrotoxic signal.

Regarding the absence of urine output data, we fully acknowledge that the KDIGO criteria recommend incorporating both SCr and urine output for a comprehensive diagnosis of AKI. However, due to the retrospective design of our study, precise urine output measurements were not consistently available across the electronic medical records of all participating centers. To preserve data integrity and ensure analytical consistency, we therefore defined AKI exclusively based on SCr changes. This limitation is explicitly addressed in the discussion section, and we agree that it may have led to an underestimation of early, mild, or oliguric AKI. Nevertheless, although mild AKI defined solely by reduced urine output without concomitant SCr changes were undetected at baseline, no differences were observed in clinical endpoints at day 30, suggesting the absence of clinically meaningful renal outcomes.

In relation to KDIGO staging, we confirm that we reported baseline AKI severity according to KDIGO stages 1 to 3, as shown in Tables 1 and 2 [1]. Stage 1 AKI accounted for 44.2% of cases. While we agree that staging is important, especially given its prognostic impact, we did not perform stratified outcome analyses by stage, as overlap weighting substantially reduced statistical power. Nonetheless, endpoints such as “lack of renal recovery at day 30”, which by definition involves persistent stage 2 or 3 AKI, did not differ between groups, arguing against a concealed severity-related effect.

Li et al. also raised an important point about the use of “modern” tubular injury biomarkers, particularly Neutrophil Gelatinase-Associated Lipocalin (NGAL), Kidney Injury Molecule-1 (KIM-1), and Interleukin-18 (IL-18), for the early detection of subclinical AKI. We fully acknowledge the growing interest in these biomarkers. However, their use in routine is constrained due to limited accessibility, high costs and the lack of internationally accepted thresholds [3]. Our study was designed to reflect real-world intensive care unit (ICU) practices, and none of the participating centers systematically collected these biomarkers. Moreover, the specificity of NGAL and IL-18 in the context of sepsis remains controversial, further complicating their interpretation in critically ill and heterogeneous ICU populations [4, 5]. Although they may be informative in selected clinical contexts, current evidence suggests that they may not be the most reliable biomarkers for detecting aminoglycoside-associated AKI in septic conditions such as urosepsis.

We fully acknowledge the well-established nephrotoxic potential of aminoglycosides, particularly when administered over prolonged periods, and agree that renal function should be closely monitored in such cases. Rather, our objective was to evaluate the clinical impact of short-term empirical aminoglycoside use in a well-defined ICU population with urosepsis, using validated critical care endpoints.

Finally, while we recognize some limitations, our findings provide meaningful real-world evidence suggesting that short-term empirical aminoglycoside use was not associated with clinically significant renal dysfunction.

## Data Availability

Not applicable.

## Abbreviations

AKI: Acute kidney injury
ICU: Intensive care unit
IL-18: Interleukin-18
KDIGO: Kidney Disease Improving Global Outcomes
KIM-1: Kidney injury molecule-1
MAKE 30: Major Adverse Kidney Events at day 30
NGAL: Neutrophil Gelatinase-Associated Lipocalin
SCr: Serum creatinine
